# Impact of a Simulation-Based Training Program on Cup Detachment During Vacuum-Assisted Vaginal Birth: Protocol for a Multicenter Quasi-Experimental Before-and-After Study

**DOI:** 10.2196/100242

**Published:** 2026-10-23

**Authors:** Maria Rosa Vila Hernandez, Sara Tameish, Ivan Hurtado, Maria Del Mar Goya Canino, Alicia Carrera I Boix, Oscar Martinez Perez

**Affiliations:** 1 Obstetrics and Gynecology Department Hospital Santa Caterina, Salt, Institut d'Assistència Sanitària (IAS) Catalunya, 90 Carrer del Dr. CastanySalt, Catalonia, 17190, Spain, +34 686267396; 2 Obstetrics and Gynaecology Department, Hospital Universitari Sant Joan de Reus, Universitat Rovira i Virgili, Reus, Catalonia, Spain; 3 Department of Obstetrics and Gynaecology, Hospital Universitari Germans Trias i Pujol, Barcelona, Catalonia, Spain; 4 "Woman & Health" Research Group (SGR-971), Germans Trias i Pujol Research Institute (IGTP), Badalona, Barcelona, Spain; 5 Department of Pediatrics, Obstetrics & Gynaecology, and Preventive Medicine and Public Health, Universitat Autònoma de Barcelona (Bellaterra), Barcelona, Spain; 6 Maternal and Fetal Medicine Research Group, Vall d’Hebron Research Institute (VHIR), Centres de Recerca de Catalunya (CERCA), Barcelona, Spain; 7 Maternal-Fetal Medicine Unit, Department of Obstetrics, Vall d’Hebron Hospital Universitari, Vall d’Hebron Barcelona Hospital Campus, Barcelona, Spain; 8 Head of Obstetrics and Gynecology Department, HM Rio, Madrid, Spain; 9 Catholic University of Murcia, Madrid, Spain

**Keywords:** operative vaginal birth, vacuum extraction, cup detachment, pop off, simulation training, quasi-experimental study, obstetric education

## Abstract

**Background:**

Safe vacuum-assisted vaginal birth depends on appropriate case selection, accurate cup placement, controlled traction, recognition of technical difficulty, and timely abandonment of an unsuccessful attempt. Simulation-based education can improve procedural performance, but evidence that vacuum-specific simulation changes patient-level clinical practice remains limited.

**Objective:**

This study aims to estimate the change in the occurrence of cup detachment (known as “pop off”) following implementation of a standardized simulation-based training program for vacuum-assisted vaginal birth. Cup detachment was designated as the single primary outcome after evaluation of the preintervention cohort but before any postintervention versus preintervention comparative analysis.

**Methods:**

This multicenter quasi-experimental before-and-after study is being conducted in 19 Spanish maternity hospitals participating in the Registro Español de Morbimortalidad Materna y Perinatal. The implemented preintervention observation period ran from November 28, 2024, through February 28, 2026. The training and washout phase was conducted during March and April 2026, followed by postintervention data collection from May through October 2026. Preintervention clinical variables are ascertained retrospectively from medical records, whereas postintervention data collection is prospective. The intervention combines preparatory theoretical material, a 1-hour face-to-face simulation workshop using a PROMPT FLEX obstetric simulator and vacuum devices, checklist-based assessment, supervised practice, feedback, and debriefing. The primary outcome will be analyzed using modified Poisson regression to estimate risk ratios and 95% CIs, accounting for hospital and protocol-defined calendar time trends. Sequential instrumentation and conversion to cesarean birth will be analyzed separately as key secondary clinical outcomes. Ethical approval was granted by the Clinical Research Ethics Committee of Girona (code 2024.070) on August 5, 2024.

**Results:**

This study was funded in November 2024. During the preintervention phase, 1155 cases were recorded in 19 hospitals; 12 were excluded according to the selection criteria, leaving 1143 eligible cases. At the verified July 2026 project update, 144 postintervention cases had been collected. Postintervention data collection is scheduled to close in October 2026; the protocol-defined comparative analysis will be performed after database lock, with publication of the main results planned for summer 2027.

**Conclusions:**

The current analysis plan was fixed before database lock and before any formal comparative postintervention versus preintervention effectiveness analysis. The protocol also defines assessment of contamination, intervention fidelity, secular trends, and the limitations inherent to a nonrandomized before-and-after evaluation.

## Introduction

### Background

Operative vaginal birth is used when birth should be expedited in the second stage of labor and can avoid the morbidity associated with cesarean birth at full cervical dilation when appropriately indicated and performed by a skilled operator [1-3]. Nevertheless, maternal and neonatal adverse events and medicolegal consequences make correct technique, supervision, and recognition of an unsuccessful attempt clinically important [1,2,4,5].

Training opportunities may be constrained by procedure volume, time pressure, supervision requirements, and the ethical limitations of learning invasive procedures by practicing on real patients [4-7]. In Spain, simulation-based obstetric training protocols have been implemented and evaluated in other emergency scenarios, such as shoulder dystocia [8-12]. Simulation offers a controlled environment in which clinicians can practice case selection, cup placement, traction, recognition of cup detachment (also known as “pop off”), and escalation without exposing patients to training-related risk [13-17].

Within the present multicenter project, the preintervention clinical phase identified cup detachment as a frequent technical event, strengthening the rationale for a standardized training program focused on correct placement and traction. The effectiveness phase therefore evaluates whether implementation of the simulation program is followed by a measurable reduction in cup detachment in routine clinical practice [18].

### Objectives

The principal comparative objective is to estimate the change in the risk of cup detachment following implementation of the simulation-based training program. The single primary outcome for the final comparative analysis is the occurrence of at least one cup detachment during an attempted vacuum-assisted vaginal birth. This outcome was designated after evaluation of the preintervention cohort but before any comparison of preintervention and postintervention outcomes. It was selected because of its direct mechanistic relationship with the technical skills targeted by the program and its observed frequency in the preintervention cohort.

Key secondary objectives are to estimate the effect of the intervention on the number of cup detachments per attempt, sequential use of a second operative instrument, and conversion to cesarean birth after an attempted vacuum extraction. Additional maternal, neonatal, procedural, and educational outcomes are described below. Sequential instrumentation and cesarean conversion are deliberately analyzed as separate outcomes because the mechanisms leading to each decision may differ.

## Methods

### Study Design and Setting

This is a multicenter quasi-experimental before-and-after evaluation of a standardized educational intervention. Maternity care in Spain is provided across hospitals with differing levels of activity, physical and human resources, and organizational characteristics [19]. Nineteen maternity hospitals in Spain participate in the Registro Español de Morbimortalidad Materna y Perinatal–associated multicenter project [20]. The study compares clinical outcomes before and after a common intervention period, with an implementation and washout interval excluded from the primary comparison. The primary comparative analysis will be restricted to hospitals contributing eligible observations in both the preintervention and postintervention periods; hospitals contributing observations in only one period will be described separately and considered only in sensitivity analyses where appropriate.

The program was implemented across participating hospitals during a common March to April 2026 window. The primary evaluation is therefore described as an uncontrolled multicenter quasi-experimental before-and-after study. The intervention is a standardized simulation-based program in vacuum-assisted vaginal birth informed by established operative birth and obstetric simulation training frameworks [21]. Because repeated observations are available over multiple calendar months before and after implementation, calendar time will be modeled to account for underlying secular trends. The study is therefore analyzed as a nonrandomized before-and-after evaluation. Calendar time is included in modeling as a prespecified trend term, but the absence of a concurrent control group means that secular trends cannot be eliminated completely and will remain an important limitation.

Protocol reporting is informed by SPIRIT (Standard Protocol Items: Recommendations for Interventional Trials) 2025 where applicable for completeness and transparency of interventional protocol items, while recognizing that SPIRIT was developed primarily for randomized trials. TIDieR (Template for Intervention Description and Replication) [21] is used for reproducible description of the training program, TREND (Transparent Reporting of Evaluations with Nonrandomized Designs) [9] for the nonrandomized intervention evaluation, and STROBE (Strengthening the Reporting of Observational Studies in Epidemiology) for relevant observational elements [10].

### Protocol Amendments and Analysis Status

This manuscript reports a protocol amendment made after evaluation of the preintervention cohort was completed but before completion of postintervention follow-up, database lock, or any formal comparison of postintervention versus preintervention outcomes. Evaluation of the preintervention data showed that cup detachment was sufficiently frequent to permit clinically interpretable assessment and, together with an independent mechanistic rationale based on the technical content of the intervention, led the investigators to designate the occurrence of at least one cup detachment as the single primary outcome for the final comparative analysis.

We acknowledge that this designation was not prospectively specified before collection or evaluation of the preintervention data. Consequently, the final analysis should not be interpreted as a fully prospectively specified confirmatory hypothesis test. Importantly, however, the outcome was designated before the investigators had examined any postintervention versus preintervention effect, corresponding risk ratio or risk difference, or statistical significance associated with the intervention. The decision therefore could not have been based on the observed direction or magnitude of the intervention effect.

The outcome hierarchy and statistical analysis plan described in this protocol will remain fixed before completion of postintervention data collection and database lock. The primary analysis will emphasize estimation of the postintervention versus preintervention risk ratio and absolute risk difference with 95% CIs rather than reliance on a binary significance threshold. The data-informed timing of primary outcome designation, including the potential for outcome selection bias and regression to the mean, will be considered explicitly when interpreting the final comparative results.

### Participants and Eligibility

The study population comprises women aged 18 years or older and their neonates whenever vacuum extraction is attempted as the intended operative vaginal birth method at a participating center during an eligible observation period. Eligibility is defined by the attempted vacuum procedure rather than by the final mode of birth. Consequently, an attempt remains eligible whether it ends in successful vacuum-assisted vaginal birth, sequential use of another operative instrument, or conversion to cesarean birth.

Eligible pregnancies are singleton pregnancies at 37 completed weeks of gestation or later with maternal consent for use of study data according to the approved protocol. Exclusion criteria are gestational age below 37 weeks, multiple pregnancy, and use of a vacuum device solely to assist fetal extraction during an already initiated cesarean birth.

### Intervention

The intervention is a standardized simulation-based program in vacuum-assisted vaginal birth informed by established operative birth and obstetric simulation training frameworks [11,12,15,22]. Preparatory theoretical material from the training manual is provided approximately 15 to 20 days before the face-to-face session [21,22]. The face-to-face workshop lasts approximately 1 hour and includes explanation of participant roles, simulator functions and limitations, workshop objectives, and the available types of obstetric vacuum devices. Participants then undertake supervised hands-on practice with feedback.

The practical component uses a PROMPT FLEX Pelvis simulator (Limbs & Things Ltd) with the assisted vaginal birth accessory, obstetric vacuum devices, gloves, lubricant, and the required supporting equipment. Instructors assess performance with a standardized checklist informed by international simulation standards. A group debriefing follows the practical exercise to review performance, identify errors, and reinforce key technical and decision-making steps. Training content covers indications and prerequisites, identification of the fetal flexion point, cup selection and correct placement, generation of negative pressure, traction along the pelvic axis coordinated with maternal effort, recognition and prevention of cup detachment, criteria for abandoning the procedure, and escalation to an alternative delivery method [1,16,17,22-24].

### Implementation, Contamination, and Fidelity

Clinical outcomes recorded during March to April 2026 are treated as part of the implementation and washout phase and are excluded from the primary before-and-after comparison. This prevents births occurring while different proportions of clinicians were being trained from being assigned unambiguously to either exposure period.

Available implementation records will be used to determine training date, attendance, the proportion of clinicians who are trained (when information is available on the baseline number of clinicians), session duration, instructor configuration, equipment used, and completion of the standardized checklist. Staff turnover, cross-coverage between services, and informal knowledge transfer will be documented when such information is available. The primary analysis will classify exposure according to center and intervention period, irrespective of individual operator attendance. These analyses will be described as exposure- or attendance-based sensitivity analyses rather than intention-to-treat or per-protocol analyses because the study is nonrandomized.

Fidelity will be summarized by center using the standardized implementation records and instructor checklists. Central review of these records will identify deviations in content, duration, equipment, and delivery. Video recording was not incorporated systematically in the implemented program and therefore cannot be introduced retrospectively as a required fidelity measure. If sufficient between-center variation exists, training coverage or fidelity will be explored as an effect modifier.

### Study Periods

The implemented preintervention observation window ran from November 28, 2024, through February 28, 2026. This is longer than the 6-month period described in an earlier manuscript version and is reported here transparently to reflect the study as implemented. The intervention and washout phase occurred during March to April 2026. Postintervention clinical outcome collection resumed after the training phase and is planned from May through October 2026 (Multimedia Appendix 1).

### Outcome Measures

The single primary outcome is the occurrence of at least one cup detachment during an attempted vacuum-assisted birth. Cup detachment is defined as unintended separation of the vacuum cup from the fetal scalp during an extraction attempt. The primary comparison is the risk of any cup detachment in the postintervention period versus the preintervention period. The timing and rationale for designation of this outcome are described under the Protocol Amendments and Analysis Status section. Although it is designated as the single primary outcome to define the hierarchy of the final comparative analysis, this designation occurred after assessment of preintervention data and is not presented as prospectively specified from study inception.

Key secondary outcomes are the number of cup detachments per vacuum attempt, sequential instrumentation with a second operative instrument, and conversion to cesarean birth after attempted vacuum extraction. Sequential instrumentation and cesarean conversion will be reported separately rather than combined into a composite primary end point.

Other maternal and procedural secondary outcomes include total instrument application time, number of instrument applications, number of traction pulls, episiotomy, perineal trauma, cervical tear requiring suturing, postpartum hemorrhage defined as estimated blood loss of at least 1000 mL, and use of general or regional anesthesia. Neonatal outcomes include Apgar score below 7 at 5 minutes, umbilical arterial pH below 7.10, shoulder dystocia, cephalohematoma or subgaleal hematoma, neonatal intensive care admission, and hyperbilirubinemia requiring phototherapy.

Educational and process outcomes include participant satisfaction, perceived usefulness, confidence, technical skills checklist performance, training coverage, and retention of knowledge or skills at 6 months where follow-up information is available. These outcomes are secondary or exploratory and will not be used to redefine the primary clinical end point.

### Covariates and Causal Adjustment

Variables collected for characterization and possible adjustment include maternal age, operator seniority, years of professional experience, prior training exposure, indication for operative vaginal birth, vacuum device type, presence of caput succedaneum, analgesia, and fetal head position. Before database lock, a clinically informed directed acyclic graph (DAG) will be used to identify a limited adjustment set from variables measured before or at the start of the vacuum attempt [25].

The primary model will remain parsimonious to avoid overfitting. Candidate baseline covariates of highest clinical priority are indication for vacuum-assisted birth, fetal head position, operator seniority and experience, and maternal age, subject to completeness and the final DAG. Variables that could lie on the causal pathway after training, including technical performance during the procedure, will not be treated as baseline confounders. For sparse secondary outcomes, model complexity will be reduced, and Firth penalized logistic regression or cluster period aggregate analyses will be considered as sensitivity approaches [3,26].

### Data Collection and Management

Clinical information is obtained from routinely recorded medical records and entered into a secure study database after deidentification. The study includes retrospective ascertainment of eligible preintervention births and prospective collection in later phases. Access is restricted to authorized members of the research team. Data quality procedures include range and consistency checks, center queries for implausible or missing values, and duplicate record review before database lock.

The analysis dataset will preserve hospital identifier, exact or grouped calendar time, intervention period, outcome definitions, prespecified covariates, and available fidelity variables. The definitive comparative analysis will begin only after closure of postintervention collection and completion of database cleaning and lock.

### Statistical Analysis

Descriptive variables will be summarized by study period and hospital. Categorical variables will be reported as counts and percentages. Continuous variables will be reported as mean and SD when approximately symmetric and as median and IQR when markedly skewed. Baseline characteristics will be described without relying on significance tests as balance diagnostics.

The primary outcome (any cup detachment) will be analyzed using modified Poisson regression with a log link to estimate the postintervention versus preintervention risk ratio and 95% CI. The main model will include hospital fixed effects and a prespecified continuous calendar month term to account for a gradual secular trend, with cluster-robust variance estimation at the hospital level and a small-sample correction because the number of hospitals is limited. Model-based marginal risks will also be used to report an adjusted absolute risk difference and 95% CI.

Because the primary outcome was designated after evaluation of the preintervention cohort, interpretation of the primary analysis will focus on the magnitude and precision of the estimated association. The postintervention versus preintervention risk ratio, adjusted absolute risk difference, and their 95% CIs will constitute the principal inferential outputs. *P* values will be reported where applicable but will not be interpreted as providing the same confirmatory evidence as a primary outcome prospectively specified before assessment of study data.

Because the intervention was implemented within a common 2-month window and there is no concurrent control group, intervention status and calendar time are necessarily related. The primary secular trend assumption is therefore a linear calendar-month trend. Sensitivity analyses will examine a quadratic time term, alternative reasonable coding of the intervention boundary, exclusion of births close to the washout boundaries, and monthly or center-period aggregate models. These analyses can test robustness to alternative time assumptions but cannot fully remove bias from unmeasured secular changes coinciding with the intervention.

Monthly preintervention cup detachment frequencies will also be examined descriptively to assess the stability of the baseline outcome over time and whether the overall preintervention rate was disproportionately influenced by a transient increase close to implementation. These descriptive analyses may help characterize temporal instability in the preintervention period but cannot exclude regression to the mean or unmeasured secular changes in the absence of a concurrent control group.

The number of cup detachments will be analyzed using Poisson or negative binomial regression according to dispersion. Other binary outcomes will generally use modified Poisson regression; rare binary outcomes will additionally be evaluated with penalized logistic regression when standard models are unstable. Continuous outcomes will use linear regression with robust standard errors or an appropriate transformation if assumptions are seriously violated. Sequential instrumentation and cesarean conversion will be analyzed and reported separately. Secondary analyses are exploratory; effect estimates and 95% CIs will be emphasized and multiplicity will be considered when interpreting nominal *P* values.

All tests will be 2-sided with α=.05. Analyses will be performed in R (R Foundation for Statistical Computing), and the exact R version and packages will be documented in the final report. Missingness will be described by variable, center, and period. Complete-case analysis will be used when missingness is minimal; multiple imputation will be considered for materially missing adjustment covariates if the missing data assumptions and available auxiliary information make this defensible.

### Sample Size and Power

All eligible births in the implemented observation periods are included. Power is therefore described using the achieved preintervention cohort and a detectable effect calculation for plausible final postintervention sample sizes.

The observed preintervention cup detachment risk was 29.5% (337/1143). As an illustrative conservative planning scenario, if the final postintervention cohort contains approximately 300 births across 19 hospitals, a design effect approximation using the observed average preperiod cluster size and an average postperiod cluster size of 15 births gives the following 80% power detectable effects at 2-sided α=.05. These calculations assume no loss from additional covariate adjustment and should be interpreted as planning approximations; final precision will be based on the achieved data and model. The corresponding detectable postintervention effects under varying intracluster correlation assumptions are presented in Table 1.

**Table 1.** Detectable postintervention cup detachment effects under varying assumptions for intracluster correlation.

Assumed intracluster correlation coefficient	Detectable postintervention cup detachment risk (%)	Absolute reduction (percentage points)	Risk ratio (95% CI)
0.03	19.4	10.1	0.66 (0.48-0.90)
0.05	18.2	11.3	0.62 (0.43-0.88)
0.10	15.9	13.6	0.54 (0.34-0.86)

If the final postintervention sample exceeds 300 births, smaller effects will be detectable; if it is lower, detectable effects will be larger. The final results paper will report the achieved sample size, event counts, intracluster correlation estimate where estimable, CI precision, and sensitivity of conclusions to clustering assumptions.

### Ethical Considerations

Ethics approval was granted by the Clinical Research Ethics Committee of Girona (code 2024.070) on August 5, 2024. Participants provide written informed consent authorizing use and sharing of study data in accordance with the approved protocol. Data are handled according to applicable data protection requirements and the Declaration of Helsinki.

The manuscript uses an introduction, methods, results, and discussion (IMRD) structure. SPIRIT is used to support completeness of protocol reporting, TIDieR to describe the intervention, TREND to guide reporting of the nonrandomized evaluation, and STROBE for relevant observational elements [9,10,21,25]. The corresponding checklists are provided as checklist files in Checklists 1–4.

## Results

### Study Status and Timeline

Clinical data collection began on November 28, 2024. The preintervention phase closed on February 28, 2026. Across 19 hospitals, 1155 cases were recorded, and 12 were excluded according to the selection criteria, leaving 1143 eligible preintervention cases. The training and washout phase was implemented during March to April 2026 across participating hospitals. Nineteen hospitals participate in the multicenter project overall.

Postintervention clinical data collection restarted after the training phase and is scheduled to close in October 2026. As of July 2026, 144 postintervention cases had been collected. Data cleaning and descriptive quality control checks have been conducted during collection, but the protocol-defined final comparative analysis will be performed only after the postintervention database is closed and locked. The main results are planned for dissemination in summer 2027.

### Intervention Delivery Status

The simulation program was delivered during March to April 2026. The training package included preparatory material, the 1-hour practical simulation session, checklist-based instructor assessment, and group debriefing. A total of 332 postsession simulation evaluation questionnaires had been collected in the project documentation, providing complementary educational process information; these questionnaire results are not the primary clinical outcome of the present protocol.

## Discussion

### Anticipated Principal Findings

The principal comparative analysis will estimate whether implementation of the standardized simulation-based training program is associated with a change in the risk of cup detachment during attempted vacuum-assisted vaginal birth. A lower postintervention cup detachment risk would be consistent with improved cup placement, traction technique, and recognition of conditions that predispose to detachment. Clinical escalation outcomes, including sequential instrumentation and cesarean conversion, are evaluated separately because they reflect both technical performance and clinical decision-making.

### Comparison With Prior Work

Simulation is well established as a method for deliberate practice and procedural skill acquisition, and vacuum-specific studies have demonstrated improvements in technical performance and retention [13,14,24,27,28]. More recent studies continue to refine training and assessment for assisted vaginal birth [29,30]. The present study extends this work by examining whether a standardized program is associated with changes in routinely observed clinical technique across multiple hospitals [31,32].

The multicenter setting is relevant because institutional experience and operative vaginal birth volume may influence outcomes [33]. Contemporary clinical guidance emphasizes correct case selection, accurate fetal head assessment, appropriate instrument application, traction technique, and timely abandonment of an unsuccessful attempt [1]. These factors informed the training program and the prespecified clinical covariates.

### Strengths and Limitations

Strengths include a multicenter national collaboration, a standardized intervention, patient-level clinical outcomes, a clearly defined washout phase, and a primary end point directly linked to the technical skill targeted by training. The revised protocol also separates technical cup detachment outcomes from sequential instrumentation and cesarean conversion, improving mechanistic interpretation.

The principal limitation is the nonrandomized before-and-after design. The absence of a concurrent control group means that secular changes in staffing, equipment, case mix, guidelines, or clinical practice occurring around March to April 2026 may be difficult to distinguish from the intervention effect. Hospital adjustment, calendar time modeling, prespecified covariates, and sensitivity analyses reduce but cannot eliminate this risk. Differential outcome ascertainment is also possible because preintervention data were collected retrospectively from medical records, whereas postintervention data are being collected prospectively. Differences in staff turnover, cross-coverage, and completeness of baseline training may also cause contamination or imprecise fidelity measurement.

Simulation also has inherent limitations in haptic fidelity. Even physical obstetric simulators cannot fully reproduce tissue compliance, pelvic constraints, force transmission, and tactile feedback encountered in clinical birth. Evidence regarding these limitations is indirect because it arises from laboratory skills training rather than obstetric vacuum simulation, and supervised clinical experience remains essential [34].

An additional limitation concerns the timing of primary outcome designation. Cup detachment was selected as the primary outcome after evaluation of the completed preintervention cohort, although before any postintervention versus preintervention comparative analysis. This prevents the primary analysis from being regarded as a fully prospectively specified confirmatory test and introduces the possibility of data-informed outcome selection. Because the observed preintervention frequency contributed to the decision, regression to the mean is also a potential alternative explanation for part of any subsequent reduction. This concern is mitigated, but not eliminated, by the large multicenter preintervention cohort, repeated monthly observations, examination of preintervention trends, hospital adjustment, calendar time modeling, and the fact that centers were not selected for unusually high cup detachment rates. Effect sizes and CIs will therefore be interpreted alongside these design limitations rather than relying on statistical significance alone.

### Future Directions and Dissemination

After database lock, the primary analysis will estimate relative and absolute changes in cup detachment, followed by the prespecified secondary and sensitivity analyses. Educational outcomes will be analyzed separately using the Kirkpatrick framework where appropriate. Results will be submitted for peer-reviewed publication, presented at scientific meetings, and communicated to participating hospitals. If the intervention is associated with clinically meaningful improvement, the standardized training materials may support broader implementation and future controlled evaluations.

### Conclusions

This protocol describes a multicenter quasi-experimental before-and-after evaluation of a standardized simulation program for vacuum-assisted vaginal birth. The revised protocol transparently designates cup detachment as the single primary outcome for the final comparative analysis while acknowledging that this designation followed evaluation of the preintervention cohort. The outcome hierarchy and statistical analysis plan are fixed before database lock and before examination of the final postintervention versus preintervention association. Interpretation will emphasize effect estimates, CIs, temporal robustness, and the limitations inherent to the data-informed outcome designation and uncontrolled before-and-after design.

## Supplementary material

10.2196/100242 Multimedia Appendix 1 Study timeline.

10.2196/100242 Checklist 1 TIDieR intervention reporting map.

10.2196/100242 Checklist 2 TREND reporting checklist.

10.2196/100242 Checklist 3 STROBE checklist.

10.2196/100242 Checklist 4 SPIRIT checklist.

## Abbreviations

DAG: directed acyclic graph
IMRD: introduction, methods, results, and discussion
SPIRIT: Standard Protocol Items: Recommendations for Interventional Trials
STROBE: Strengthening the Reporting of Observational Studies in Epidemiology
TIDieR: Template for Intervention Description and Replication
TREND: Transparent Reporting of Evaluations with Nonrandomized Designs
