# Supplementary material for: Impact of a Simulation-Based Training Program on Cup Detachment During Vacuum-Assisted Vaginal Birth: Protocol for a Multicenter Quasi-Experimental Before-and-After Study
Source: JMIR Res Protoc. 2026 Oct 23;15:e100242. doi: 10.2196/100242 (PMC13693959; doi:10.2196/100242)
Supplement: Supplementary file 1 [file resprot-v15-e100242-s001.docx]

# Appendix 1. Study Timeline

| **Phase** | **Implemented / planned dates** | **Status** |
| --- | --- | --- |
| Pre-intervention clinical collection | November 28, 2024-February 28, 2026 | Completed; 1,143 eligible cases from 19 hospitals |
| Intervention / washout | March-April 2026 | Completed |
| Post-intervention clinical collection | May-October 2026 | In progress at July 2026 update; 144 cases collected |
| Database lock and final comparative analysis | After October 2026 | Planned |
| Main dissemination | 2027 | Planned |

Clinical observations from the intervention/washout phase are excluded from the primary before-and-after comparison.
