# Supplementary material for: Impact of a Simulation-Based Training Program on Cup Detachment During Vacuum-Assisted Vaginal Birth: Protocol for a Multicenter Quasi-Experimental Before-and-After Study
Source: JMIR Res Protoc. 2026 Oct 23;15:e100242. doi: 10.2196/100242 (PMC13693959; doi:10.2196/100242)
Supplement: Supplementary file 2 [file resprot-v15-e100242-s002.docx]

# Appendix 2. TIDieR Intervention Reporting Map

| **TIDieR domain** | **Study specification** |
| --- | --- |
| Brief name | Standardised simulation-based training programme in vacuum-assisted vaginal birth |
| Why | Improve technical execution of vacuum-assisted birth and reduce cup detachment |
| Materials | Training manual material; PELVIS PROMPT FLEX simulator with AVB accessory; vacuum devices; gloves; lubricant; technical-skills checklist |
| Procedures | Preparatory theory; one-hour face-to-face simulation; supervised practice; checklist assessment; expert feedback; group debriefing |
| Who provided | Experienced obstetric instructors / locally trained instructors |
| How | Face-to-face simulation supported by preparatory theoretical material |
| Where | Participating Spanish maternity hospitals |
| When/how much | Theoretical material approximately 15-20 days before; approximately one-hour face-to-face workshop; intervention delivered March-April 2026 |
| Tailoring | Core educational content standardised; local scheduling and logistics adapted to each centre |
| How well - planned | Attendance, duration, equipment, checklist completion, proportion of clinicians trained when denominators are available |
| How well - actual | Will be reported by centre in the final results paper; central review of available logs and checklists |
