# Supplementary material for: Impact of a Simulation-Based Training Program on Cup Detachment During Vacuum-Assisted Vaginal Birth: Protocol for a Multicenter Quasi-Experimental Before-and-After Study
Source: JMIR Res Protoc. 2026 Oct 23;15:e100242. doi: 10.2196/100242 (PMC13693959; doi:10.2196/100242)
Supplement: Supplementary file 3 [file resprot-v15-e100242-s003.docx]

# Appendix 3. TREND Reporting Checklist / Protocol Reporting Map

| **TREND domain** | **Status** | **How addressed in this protocol** |
| --- | --- | --- |
| Title and abstract | Addressed | The title identifies a multicentre quasi-experimental before-and-after study; the abstract states the intervention, primary outcome, study periods, analysis strategy, ethics approval, and current study status. |
| Background and rationale | Addressed | Introduction - Background: clinical relevance of operative vaginal birth, limitations of workplace training, rationale for simulation, and evidence gap regarding transfer to patient-level outcomes. |
| Study design | Addressed | Methods - Study Design and Setting: nonrandomised multicentre before-and-after evaluation with a common intervention/washout period and no concurrent control group. |
| Participants and eligibility | Addressed | Methods - Participants and Eligibility: women aged 18 years or older with attempted vacuum extraction; singleton pregnancy at 37 completed weeks or later; prespecified exclusions are stated. |
| Intervention description | Addressed | Methods - Intervention and Appendix 2: preparatory theory, one-hour face-to-face simulation, PROMPT FLEX simulator, supervised practice, checklist-based assessment, feedback, and debriefing. |
| Implementation, exposure, and fidelity | Addressed | Methods - Implementation, Contamination, and Fidelity: attendance, coverage, session duration, instructors, equipment, checklist completion, staff turnover, cross-coverage, and deviations will be summarised where available. |
| Objectives and hypotheses | Addressed | Methods - Objectives: primary objective is estimate the change in the risk of at least one cup detachment (pop-off); secondary procedural, clinical, and educational objectives are specified. |
| Outcome measures | Addressed | Methods - Outcome Measures: single binary primary outcome (any pop-off) plus prespecified secondary maternal, neonatal, procedural, and educational outcomes. |
| Sample size / precision | Addressed | Methods - Sample Size and Power: all eligible births are included; detectable-effect calculations are presented as planning approximations, with final interpretation based on achieved sample size and confidence-interval precision. |
| Assignment method | Not applicable | Centres were not randomised and there was no staggered randomised rollout. Exposure is defined by the common pre-intervention and post-intervention periods. |
| Masking / blinding | Not applicable / not feasible | The educational intervention and clinical period are not maskable. |
| Unit of analysis and clustering | Addressed | Methods - Statistical Analysis: patient-level outcomes are analysed with hospital fixed effects and hospital-clustered variance estimation with a prespecified small-sample correction. |
| Statistical methods | Addressed | Methods - Statistical Analysis: modified Poisson regression for the primary risk ratio, calendar-time adjustment, marginal absolute risk difference, sensitivity analyses for time assumptions, and methods for secondary outcomes and missing data. |
| Participant flow and recruitment dates | Partly addressed; final flow pending | Results - Study Status and Timeline: pre-intervention recruitment, exclusions, eligible cases, intervention/washout dates, and current post-intervention recruitment are reported. Final post-intervention flow will be reported after database lock. |
| Baseline characteristics | Planned | Methods state that characteristics will be summarised by study period and hospital without significance testing as a balance diagnostic. |
| Numbers analysed and outcome estimates | Planned | The final results report will provide denominators, event counts, relative and absolute effect estimates, 95% CIs, and sensitivity analyses after database lock. |
| Adverse / unintended effects | Planned | Maternal and neonatal adverse outcomes are prespecified as secondary outcomes; clinically relevant unintended effects will be reported in the final analysis where captured. |
| Interpretation and limitations | Addressed at protocol stage | Discussion - Strengths and Limitations: absence of a concurrent control group, secular trends, contamination, fidelity limitations, staff turnover, and differential ascertainment from retrospective PRE versus prospective POST collection are acknowledged. |
| Generalisability | Addressed | Discussion notes the multicentre setting and the relevance of institutional experience and operative vaginal birth volume; external validity will be interpreted in light of participating hospitals and training coverage. |
| Ethics and dissemination | Addressed | Methods - Ethics and Reporting Guidance and Discussion - Future Directions and Dissemination: ethics approval, consent according to the approved protocol, data protection, publication, meeting presentation, and feedback to participating hospitals are described. |
