# Supplementary material for: Impact of a Simulation-Based Training Program on Cup Detachment During Vacuum-Assisted Vaginal Birth: Protocol for a Multicenter Quasi-Experimental Before-and-After Study
Source: JMIR Res Protoc. 2026 Oct 23;15:e100242. doi: 10.2196/100242 (PMC13693959; doi:10.2196/100242)
Supplement: Supplementary file 4 [file resprot-v15-e100242-s004.docx]

# Appendix 4. STROBE Checklist / Observational Reporting Map

STROBE is used here for the observational components of the before-and-after evaluation. As this is a protocol manuscript, items requiring completed outcome analyses are marked as planned for the final results paper.

| **STROBE item / domain** | **Status** | **How addressed in this protocol** |
| --- | --- | --- |
| Title and abstract | Addressed | The design is identified in the title and abstract, and the abstract summarises setting, intervention, primary outcome, study periods, analysis plan, ethics approval, and study status. |
| Background / rationale | Addressed | Introduction explains the clinical importance of safe vacuum-assisted birth, limitations of experiential training, the role of simulation, and the limited evidence for patient-level effects. |
| Objectives | Addressed | The primary and secondary objectives are explicitly stated, with any pop-off defined as the single primary outcome. |
| Study design | Addressed | Methods identifies a multicentre quasi-experimental before-and-after evaluation, with an excluded implementation/washout period and no concurrent control group. |
| Setting | Addressed | Ninteen Spanish maternity hospitals participating in the REMMP-associated multicentre project are described; 19 contributed eligible pre-intervention cases. |
| Participants | Addressed | Eligibility is based on attempted vacuum extraction rather than final mode of birth; inclusion and exclusion criteria are specified. |
| Variables | Addressed | Primary, secondary, educational/process outcomes and candidate covariates are defined in Methods - Outcome Measures and Covariates and Causal Adjustment. |
| Data sources and measurement | Addressed | Clinical data are obtained from medical records and entered into a secure de-identified database; identical operational definitions are intended across periods, with range, consistency, duplicate, and centre-query checks. |
| Bias | Addressed | The protocol addresses secular trends, hospital-level differences, contamination, implementation fidelity, staff turnover, cross-coverage, and possible differential ascertainment because PRE is retrospectively ascertained whereas later phases are prospectively collected. |
| Study size | Addressed | All eligible births in the implemented observation periods are included. The protocol provides detectable-effect / precision calculations based on the achieved pre-intervention cohort and plausible post-intervention sample sizes. |
| Quantitative variables | Addressed | Continuous variables will be summarised according to distribution. Calendar time is prespecified as a continuous month term in the primary model, with alternative time specifications examined in sensitivity analyses. |
| Statistical methods - confounding | Addressed | Hospital fixed effects, calendar time, and a limited clinically informed adjustment set based on variables measured before or at the start of the vacuum attempt are specified. |
| Statistical methods - clustering | Addressed | The primary analysis accounts for hospital clustering using cluster-robust variance estimation with a small-sample correction; aggregate centre-period analyses are planned as sensitivity analyses. |
| Statistical methods - missing data | Addressed | Missingness will be described by variable, centre, and period; complete-case analysis is planned when missingness is minimal, with multiple imputation considered for materially missing adjustment covariates when assumptions are defensible. |
| Statistical methods - sensitivity analyses | Addressed | Alternative time trends, intervention boundaries, exclusion around washout boundaries, aggregate analyses, and alternative models for sparse outcomes are prespecified. |
| Participants / flow | Partly addressed; final flow pending | Results report 1,155 recorded PRE cases, 12 exclusions, and 1,143 eligible cases from 19 hospitals; final POST flow and analysed numbers will be reported after closure and database lock. |
| Descriptive data | Planned | Maternal, operator, procedural, and centre characteristics will be reported by period and hospital; missingness will be described. |
| Outcome data | Partly addressed; comparative results pending | The observed PRE pop-off risk is reported for planning purposes. Final POST outcome frequencies and comparative estimates will only be reported after database lock. |
| Main results | Planned | The final paper will report adjusted and unadjusted effect estimates as appropriate, with risk ratios, absolute risk differences, and 95% confidence intervals. |
| Other analyses | Planned | Secondary, fidelity, training-coverage, time-trend, boundary, and sparse-outcome sensitivity analyses are prespecified. |
| Key results | Protocol-stage discussion | Discussion describes the planned principal comparative analysis without claiming an intervention effect before completion of the comparative analysis |
| Limitations | Addressed | The absence of a concurrent control group, residual secular confounding, fidelity measurement, contamination, staff changes, haptic limitations of simulation, and differential data ascertainment are acknowledged. |
| Interpretation | Addressed | The protocol states that any observed post-intervention reduction will be interpreted as an association consistent with improved technique, while recognising that causal attribution is limited by the nonrandomised design. |
| Generalisability | Addressed | The multicentre Spanish setting is described and the influence of institutional experience, procedure volume, participation, and training coverage will be considered when interpreting external validity. |
| Funding | Addressed | The manuscript states that there is no funding. |
