# Supplementary material for: Impact of a Simulation-Based Training Program on Cup Detachment During Vacuum-Assisted Vaginal Birth: Protocol for a Multicenter Quasi-Experimental Before-and-After Study
Source: JMIR Res Protoc. 2026 Oct 23;15:e100242. doi: 10.2196/100242 (PMC13693959; doi:10.2196/100242)
Supplement: Supplementary file 5 [file resprot-v15-e100242-s005.docx]

**SPIRIT 2025 Checklist**

**Impact of a Simulation-Based Training Program on Cup Detachment During Vacuum-Assisted Vaginal Birth: Protocol for a Multicenter Quasi-Experimental Before-and-After Study**

Note: SPIRIT 2025 was developed primarily for randomized trials. This study is a nonrandomized multicenter quasi-experimental before-and-after evaluation; therefore, randomization, allocation concealment, blinding, and other trial-specific items are marked as not applicable where appropriate. Page numbers refer to the current 9-page proof.

| **Section / topic** | **No.** | **SPIRIT 2025 checklist item** | **Reported on page / section in manuscript** |
| --- | --- | --- | --- |
| Administrative information | 1a | Title stating the trial design, population, and interventions, with identification as a protocol | Page 1 — Title. |
| Administrative information | 1b | Structured summary of trial design and methods, including items from the World Health Organization Trial Registration Data Set | Pages 1–2 — Abstract (Background, Objective, Methods, Results, Conclusions, IRRID). |
| Administrative information | 2 | Version date and identifier | Not reported as a separate protocol version/date in the manuscript. |
| Administrative information | 3a | Names, affiliations, and roles of protocol contributors | Page 1 — Authors and affiliations; page 7 — Authors’ Contributions. |
| Administrative information | 3b | Name and contact information for the trial sponsor | Not applicable / no sponsor identified. Page 7 states that no financial support was received. |
| Administrative information | 3c | Role of trial sponsor and funders in design, conduct, analysis, and reporting of trial; including any authority over these activities | Not applicable — no sponsor or funder. Page 7 — Funding. |
| Administrative information | 3d | Composition, roles, and responsibilities of the coordinating site, steering committee, endpoint adjudication committee, data management team, and other individuals or groups overseeing the trial, if applicable | No formal steering, adjudication, or data monitoring committees are described. Relevant implementation oversight is described on page 3 under Implementation, Contamination, and Fidelity. |
| Open science | 4 | Name of trial registry, identifying number (with URL), and date of registration. If not yet registered, name of intended registry | Not reported as a clinical trial registration. The manuscript provides an IRRID (page 2), which is a publication identifier rather than a trial registry number. |
| Open science | 5 | Where the trial protocol and statistical analysis plan can be accessed | The present article is the study protocol. Statistical analysis plan: pages 4–5 — Statistical Analysis. |
| Open science | 6 | Where and how the individual de-identified participant data (including data dictionary), statistical code, and any other materials will be accessible | Page 7 — Data Availability. |
| Open science | 7a | Sources of funding and other support (eg, supply of drugs) | Page 7 — Funding: no financial support received. |
| Open science | 7b | Financial and other conflicts of interest for principal investigators and steering committee members | Page 7 — Conflicts of Interest: none declared. |
| Open science | 8 | Plans to communicate trial results to participants, healthcare professionals, the public, and other relevant groups (eg, reporting in trial registry, plain language summary, publication) | Page 6 — Future Directions and Dissemination; page 7 — Data Availability. |
| Introduction | 9a | Scientific background and rationale, including summary of relevant studies (published and unpublished) examining benefits and harms for each intervention | Page 2 — Introduction, Background. |
| Introduction | 9b | Explanation for choice of comparator | Adapted to the nonrandomized design: page 2 — Study Design and Setting. The postintervention period is compared with the preintervention period; no randomized comparator is used. |
| Introduction | 10 | Specific objectives related to benefits and harms | Page 2 — Objectives. |
| Methods: Patient and public involvement, trial design | 11 | Details of, or plans for, patient or public involvement in the design, conduct, and reporting of the trial | Not reported in the manuscript. |
| Methods: Patient and public involvement, trial design | 12 | Description of trial design including type of trial (eg, parallel group, crossover), allocation ratio, and framework (eg, superiority, equivalence, non-inferiority, exploratory) | Page 2 — Study Design and Setting: uncontrolled multicenter quasi-experimental before-and-after evaluation. |
| Methods: Participants, interventions, and outcomes | 13 | Settings (eg, community, hospital) and locations (eg, countries, sites) where the trial will be conducted | Pages 1–2 — 19 maternity hospitals in Spain; Study Design and Setting. |
| Methods: Participants, interventions, and outcomes | 14a | Eligibility criteria for participants | Page 3 — Participants and Eligibility. |
| Methods: Participants, interventions, and outcomes | 14b | If applicable, eligibility criteria for sites and for individuals who will deliver the interventions (eg, surgeons, physiotherapists) | Not explicitly reported. Participating maternity hospitals and instructors are described, but formal eligibility criteria for sites/instructors are not specified. |
| Methods: Participants, interventions, and outcomes | 15a | Intervention and comparator with sufficient details to allow replication including how, when, and by whom they will be administered. If relevant, where additional materials describing the intervention and comparator (eg, intervention manual) can be accessed | Page 3 — Intervention; page 3 — Implementation, Contamination, and Fidelity. Training materials/manuals are referenced in the manuscript. |
| Methods: Participants, interventions, and outcomes | 15b | Criteria for discontinuing or modifying allocated intervention/comparator for a trial participant (eg, drug dose change in response to harms, participant request, or improving/worsening disease) | Not applicable — this is a center-level educational intervention with no participant-level allocated treatment. |
| Methods: Participants, interventions, and outcomes | 15c | Strategies to improve adherence to intervention/comparator protocols, if applicable, and any procedures for monitoring adherence (eg, drug tablet return, sessions attended) | Page 3 — Implementation, Contamination, and Fidelity: attendance, training coverage, checklist completion, session duration, instructor configuration, and fidelity records. |
| Methods: Participants, interventions, and outcomes | 15d | Concomitant care that is permitted or prohibited during the trial | Not applicable — routine obstetric care is not restricted by the educational intervention. |
| Methods: Participants, interventions, and outcomes | 16 | Primary and secondary outcomes, including the specific measurement variable, analysis metric, method of aggregation, and time point for each outcome | Pages 4–5 — Outcome Measures and Statistical Analysis. |
| Methods: Participants, interventions, and outcomes | 17 | How harms are defined and will be assessed (eg, systematically, non-systematically) | Page 4 — Outcome Measures lists maternal and neonatal adverse outcomes and their definitions where applicable; page 4 — Data Collection and Management describes ascertainment from clinical records. No separate training-related harm assessment is specified. |
| Methods: Participants, interventions, and outcomes | 18 | Time schedule of enrolment, interventions (including any run-ins and washouts), assessments, and visits for participants. A schematic diagram is highly recommended | Page 4 — Study Periods; page 5 — Study Status and Timeline; Checklist 1 — Study timeline. |
| Methods: Participants, interventions, and outcomes | 19 | How sample size was determined, including all assumptions supporting the sample size calculation | Page 5 — Sample Size and Power; Table 1. |
| Methods: Participants, interventions, and outcomes | 20 | Strategies for achieving adequate participant enrolment to reach target sample size | Adapted to study design: page 5 — Sample Size and Power states that all eligible births in the implemented observation periods are included; no separate recruitment target is used. |
| Methods: Assignment of interventions | 21a | Who will generate the random allocation sequence and the method used | Not applicable — nonrandomized before-and-after design. |
| Methods: Assignment of interventions | 21b | Type of randomisation (simple or restricted) and details of any factors for stratification | Not applicable — nonrandomized before-and-after design. |
| Methods: Assignment of interventions | 22 | Mechanism used to implement the random allocation sequence, describing any steps to conceal the sequence until interventions are assigned | Not applicable — no random allocation. |
| Methods: Assignment of interventions | 23 | Whether the personnel who will enrol and those who will assign participants to the interventions will have access to the random allocation sequence | Not applicable — no random allocation. |
| Methods: Assignment of interventions | 24a | Who will be blinded after assignment to interventions (eg, participants, care providers, outcome assessors, data analysts) | Not applicable — no randomized assignment and no blinding described. |
| Methods: Assignment of interventions | 24b | If blinded, how blinding will be achieved and description of the similarity of interventions | Not applicable. |
| Methods: Assignment of interventions | 24c | If blinded, circumstances under which unblinding is permissible, and procedure for revealing a participant’s allocated intervention during the trial | Not applicable. |
| Methods: Data collection, management, and analysis | 25a | Plans for assessment and collection of trial data, including processes to promote data quality and a description of trial instruments, with reference to data collection forms if available | Page 4 — Outcome Measures; page 4 — Data Collection and Management. |
| Methods: Data collection, management, and analysis | 25b | Plans to promote participant retention and complete follow-up, including outcome data to be collected for participants who discontinue or deviate from intervention protocols | Not applicable to the primary clinical before-and-after evaluation; outcomes are obtained from routinely recorded clinical data rather than participant follow-up. |
| Methods: Data collection, management, and analysis | 26 | Plans for data entry, coding, security, and storage, including processes to promote data quality. Reference to where details of data management procedures can be accessed, if not in the protocol | Page 4 — Data Collection and Management. |
| Methods: Data collection, management, and analysis | 27a | Statistical methods used to compare groups for primary and secondary outcomes, including harms | Pages 4–5 — Statistical Analysis. |
| Methods: Data collection, management, and analysis | 27b | Definition of who will be included in each analysis (eg, all randomised participants), and in which group | Page 2 — Study Design and Setting (primary analysis restricted to hospitals contributing eligible observations in both periods); page 3 — Implementation, Contamination, and Fidelity (exposure classified by center and intervention period). |
| Methods: Data collection, management, and analysis | 27c | How missing data will be handled in the analysis | Page 5 — Statistical Analysis. |
| Methods: Data collection, management, and analysis | 27d | Methods for any additional analyses (eg, subgroup and sensitivity analyses) | Pages 4–5 — Covariates and Causal Adjustment; Statistical Analysis; sensitivity analyses. |
| Methods: Monitoring | 28a | Composition of data monitoring committee (DMC); summary of its role and reporting structure; statement of independence and conflicts of interest; or explanation of why a DMC is not needed | Not applicable / no DMC described. This is a nonrandomized educational intervention using routine clinical data. |
| Methods: Monitoring | 28b | Explanation of any interim analyses and stopping guidelines, including who will have access to interim results and make the final decision to terminate the trial | Not applicable. Page 6 states that the protocol-defined final comparative analysis will be performed only after postintervention database closure and lock. |
| Methods: Monitoring | 29 | Frequency and procedures for monitoring trial conduct. If there is no monitoring, give explanation | Pages 3–4 — Implementation, Contamination, and Fidelity; Data Collection and Management. Fidelity records, central review, range/consistency checks, center queries, and duplicate review are described. |
| Ethics | 30 | Plans for seeking research ethics committee/institutional review board approval | Page 5 — Ethical Considerations; approval granted by the Clinical Research Ethics Committee of Girona (code 2024.070). |
| Ethics | 31 | Plans for communicating important protocol modifications to relevant parties | Page 3 — Protocol Amendments and Analysis Status describes the protocol amendment and its timing. A general communication plan for future amendments is not separately specified. |
| Ethics | 32a | Who will obtain informed consent or assent from potential trial participants or authorised proxies, and how | Page 5 — Ethical Considerations states that participants provide written informed consent. The specific personnel obtaining consent are not stated. |
| Ethics | 32b | Additional consent provisions for collection and use of participant data and biological specimens in ancillary studies, if applicable | Not applicable — no ancillary biological specimen studies are described. |
| Ethics | 33 | How personal information about potential and enrolled participants will be collected, shared, and maintained to protect confidentiality before, during, and after the trial | Page 4 — Data Collection and Management: deidentification, secure database, restricted access; page 7 — Data Availability. |
| Ethics | 34 | Provisions, if any, for ancillary and post-trial care, and for compensation to those who suffer harm from trial participation | Not applicable to this nonrandomized educational intervention; no participant-level experimental treatment is assigned and no specific compensation provisions are reported. |

*Checklist source: SPIRIT 2025 (Standard Protocol Items: Recommendations for Interventional Trials), Chan et al., 2025. The checklist is applied here only where relevant to this nonrandomized quasi-experimental protocol.*
